# Overt Hypothyroidism Status Post Pfizer-BioNTech Vaccination: A Case Study

**DOI:** 10.7759/cureus.41180

**Published:** 2023-06-30

**Authors:** Sailaja Nandennagari, Preethi Annam, Nithish Naidu, Prakhya Vegesna, Krupavaram Bethala

**Affiliations:** 1 Medicine, Avalon University School of Medicine, Willemstad, CUW; 2 Surgery, Avalon University School of Medicine, Willemstad, CUW; 3 Family Medicine, American University of Barbados, Wildey, BRB; 4 Pharmacology and Therapeutics, Centre of Excellence in Pharmaceutical Sciences, School of Pharmacy, KPJ Healthcare University College, Nilai, MYS

**Keywords:** covid-19 vaccine adverse effects, covid-19 vaccine side effects, covid 19 vaccination, vaccination, hypothyroidism, pfizer, pandemic, covid-19

## Abstract

Coronavirus disease (COVID-19) is among the most contagious viral illnesses, affecting millions worldwide. Although precautions such as social distancing, hand sanitizing, and the use of masks decreased the transmission of the virus, the situation went uncontrolled until vaccination came to light. Vaccination was vital in limiting the incidence, prevalence, and severity caused by the severe acute respiratory syndrome coronavirus 2 (SARS-CoV-2). Based on the mechanism, several types of vaccines, such as Pfizer-BioNTech, Moderna, AstraZeneca, Johnson & Johnson, and Covaxin, were approved by the US Food and Drug Administration (FDA). A booster dose was implemented as the vaccine's effectiveness decreased with time.

Several side effects, such as fever, soreness around the injection site, fatigue, chills, muscle weakness, and headache, have been reported after vaccination with Pfizer-BioNTech, but thyroid dysfunction is relatively rare. Several case reports and even case series describing links between COVID-19 vaccination and various types of thyroid dysfunction have appeared in the literature. However, the exact reasons have yet to be explained. This report presents the case of a healthy 50-year-old woman diagnosed with overt hypothyroidism three weeks after the administration of the Pfizer-BioNTech vaccine.

## Introduction

Due to the global COVID-19 pandemic, approximately 761 million cases and six million deaths have been reported as of March 31, 2023 [1]. Although there is no definitive cure for COVID-19, the advent of vaccines has played a vital role in developing immunity to the virus and reducing the risk of severe complications and death. As with any treatment or prevention method, vaccination has side effects. Aside from the many different side effects that people experience from vaccines, hypothyroidism is also a side effect that occurs due to a thyroid hormone deficiency that affects multiple organ systems. Thyroid hormones regulate critical activities such as metabolism, blood formation, and tissue growth. Low free thyroxine (T4) levels can distinguish overt hypothyroidism from asymptomatic types. This is a clinical case report of a healthy woman who developed hypothyroidism three weeks after her first dose of the Pfizer-BioNTech COVID-19 vaccine.

## Case presentation

A 50-year-old Caucasian woman with an unremarkable medical history presented to the hospital complaining of palpitations, fatigue, constipation, and weakness. The patient noticed a weight gain of seven pounds in six weeks since the first dose of the vaccine. The patient said she had these symptoms two weeks after getting the Pfizer-BioNTech vaccine. She said that she was in perfect health, had been well up to that point, and had not needed any medication in her daily life. The family history was negative for thyroid and autoimmune disorders. The patient stated that her last menstrual period was two weeks ago. The patient's vital signs were recorded: height: 5.3 feet, weight: 188 lbs, body mass index: 31 kg/m2, heart rate: 66 beats/min, and blood pressure: 142/80 mmHg. A physical examination revealed dry skin and slow reflexes. No sounds were auscultated. Tests for anti-thyroglobulin (TG), anti-thyroid peroxidase (TPO), and antinuclear antibodies (ANA) were negative. The patient's laboratory test results displaying overt hypothyroid status are in Table 1.

**Table 1:** The patient's laboratory results display overt hypothyroid status after COVID-19 messenger RNA (mRNA) vaccination

Thyroid hormones	Laboratory findings	Reference values
Free triiodothyronine (T3)	100 pg/dL	130 to 450 pg/dL
Free thyroxine (T4)	0.5 ng/dL	0.9 to 2.3 ng/dL
Thyroid-stimulating hormone (TSH)	10.6 mU/L	0.4 to 4.0 mU/L

The patient was started on 25 mg of levothyroxine. 

## Discussion

The COVID-19 pandemic, which began in December 2019, was caused by SARS-CoV-2 and resulted in the advent of various vaccines for its prevention. Several viral vectors (genetic, inactivated, attenuated, and protein vaccines) have been developed to overcome the devastating COVID-19 pandemic. The World Health Organization (WHO) has approved mRNA-based vaccines such as Moderna and Pfizer-BioNTech, inactivated vaccines such as Covaxin, and viral vector vaccines such as those from AstraZeneca and Johnson & Johnson. Based on our literature search, we found that Jafarzadeh et al. documented various types of thyroid dysfunction post Pfizer-BioNTech vaccine; the prevalence of overt hypothyroidism in their study was 1.2% [2]. We found a similar article describing overt hypothyroidism in a 61-year-old woman post-mRNA vaccine [3]. The average side effects of fatigue, headache, fever, and muscle weakness after the first dose of Pfizer-BioNTech's COVID vaccine were 79%, compared with 84% after the second dose. Even though there is ample literature on side effects from Pfizer-BioNTech vaccinations, studies on hypothyroidism after the vaccination are minimal. The average rate of side effects in women is 69.8%, compared to 30.2% in men [4]. SARS-CoV-2 may directly affect thyroid function by using the angiotensin-converting enzyme 2 (ACE2) as a receptor for host cell entry expressed by thyroid follicular cells. Severe COVID-19 infection triggers a systemic immune and inflammatory response that triggers the release of solid pathogenic cytokines such as interleukin-1 beta (IL-1β), interleukin-6 (IL-6), tumor necrosis factor-alpha (TNF-α), and interferon-gamma (IFN‐γ), ultimately leading to multiple organ damage [5].

Pfizer-BioNTech is an mRNA-based vaccine using various lipids, including polyethylene glycol, that can trigger immune responses leading to anaphylaxis [6]. The exact mechanism of hypothyroidism after COVID-19 vaccination is still unknown. Differentials must include subacute thyroiditis, silent thyroiditis, and focal painful thyroiditis. Including autoimmune hypothyroidism in surveillance following COVID-19 immunization is essential, apart from only autoimmune hyperthyroidism or subacute thyroiditis. After receiving a vaccine, the above-mentioned thyroid dysfunctions may appear within a few weeks. We firmly advise surveillance and safety monitoring of more authorized candidate vaccines in the future.

## Conclusions

The global COVID-19 pandemic is more effectively controlled and managed through vaccination, as no definitive treatment covers all short- and long-term signs and symptoms of infection. The COVID-19 vaccination is effective, but it has side effects like any treatment. With the COVID-19 vaccine, about one in six people may experience side effects after being vaccinated. The Pfizer-BioNTech vaccines appear to have the fewest side effects among other vaccines. However, autoimmune diseases such as overt hypothyroidism have been reported as one specific side effect experienced by patients, such as those experienced by patients using the Pfizer-BioNTech vaccine. The vaccine's mechanism of causing hypothyroidism is still being studied, but vaccines continue to improve, and the benefits outweigh the side effects. This clinical inquiry may progress to study the exact mechanism associated with the Pfizer-BioNTech vaccine and hypothyroidism.

## References

[1] (2023). WHO coronavirus (COVID-19) dashboard. https://covid19.who.int/.

[2] Jafarzadeh A, Nemati M, Jafarzadeh S, Nozari P, Mortazavi SM (2022). Thyroid dysfunction following vaccination with COVID-19 vaccines: a basic review of the preliminary evidence. J Endocrinol Invest.

[3] Giusti M, Maio A (2021). Acute thyroid swelling with severe hypothyroid myxoedema after COVID-19 vaccination. Clin Case Rep.

[4] Dighriri IM, Alhusayni KM, Mobarki AY (2022). Pfizer-BioNTech COVID-19 vaccine (BNT162b2) side effects: a systematic review. Cureus.

[5] Ruggeri RM, Campennì A, Deandreis D, Siracusa M, Tozzoli R, Petranović Ovčariček P, Giovanella L (2021). SARS-COV-2-related immune-inflammatory thyroid disorders: facts and perspectives. Expert Rev Clin Immunol.

[6] Khan F, Brassill MJ (2021). Subacute thyroiditis post-Pfizer-BioNTech mRNA vaccination for COVID-19. Endocrinol Diabetes Metab Case Rep.

